# Validation of the Oncofertility Support Scale derived from the Fertility Information Support scale among young adult women with breast cancer

**DOI:** 10.1007/s00520-025-10250-0

**Published:** 2025-12-19

**Authors:** Li Hu, Pui Hing Chau, Chanchan Wu, Edmond Pui Hang Choi

**Affiliations:** 1 School of Nursing, LKS Faculty of Medicine, The University of Hong Kong, 5/F, Academic Building, 3 Sassoon Road Pokfulam, Hong Kong, China; 2 Breast Surgical Department, The Affiliated Hospital of Guizhou Medical University, Guiyang, China; 3 School of Nursing, Faculty of Health and Social Sciences, The Hong Kong Polytechnic University, Hong Kong, China

**Keywords:** Carcinoma, Breast cancer, Women, Oncofertility support

## Abstract

**Purpose:**

Oncofertility support plays a critical role in coping with fertility issues among women with breast cancer. There is a growing demand for reliable tools to assess and enhance oncofertility support. This study aimed to validate a tool assessing oncofertility support.

**Methods:**

The study involved two phases. First, insights from healthcare professionals and women with breast cancer were used to modify the Fertility Information Support Scale, resulting in a 22-item Oncofertility Support Scale. Second, a cross-sectional study was conducted among 343 participants aged 18–40 to assess the scale’s content validity, structural validity, measurement invariance, convergent validity, known-group validity, and internal consistency reliability.

**Results:**

Exploratory factor analysis yielded a 19-item scale across four dimensions: information support on fertility impact, information support on fertility preservation, fertility and sexual guidance, and fertility communication and supportive networks. Confirmatory factor analysis confirmed the scale’s structure. Multi-group confirmatory factor analysis showed measurement invariance across age and fertility desire groups. The content validity for the scale was 0.95. Oncofertility support showed a weak association with medical social support. Average Variance Extracted (AVE ≥ 0.63) and Composite Reliability (CR ≥ 0.90) supported the convergent validity of the scale. Women aged ≤ 30 reported higher information support on fertility preservation and fertility communication and supportive networks than those older than 30. Both Cronbach’s alpha and McDonald’s ω were 0.95.

**Conclusion:**

The Oncofertility Support Scale is a valid and reliable measure for assessing oncofertility support among women with breast cancer.

**Supplementary Information:**

The online version contains supplementary material available at 10.1007/s00520-025-10250-0.

## Introduction

Breast cancer is the most common cancer among women [1]. Advances in treatment have significantly increased survival rates, with the five-year survival rate reaching 90% [2]. As survival improves, clinical attention has increasingly shifted toward the long-term physical and psychosocial challenges experienced during survivorship [3]. Among young women with breast cancer, fertility has become a critical concern [4]. Chemotherapy can lead to temporary or permanent infertility, and long-term endocrine therapy may cause young women to miss the optimal reproductive window, thereby reducing their chances of conceiving [5]. Fertility issues are particularly pronounced among women carrying pathogenic variants in BRCA1 or BRCA2, who face the dual challenge of potential transmission of pathogenic variants to offspring and accelerated decline in ovarian reserve due to the biological role of BRCA genes. Risk-reducing bilateral salpingo-oophorectomy performed for ovarian cancer prevention further complicates fertility decision-making, creating complex fertility challenges for young women with breast cancer [6].

Given the societal trend of delayed marriage and childbearing, many women have not yet completed family planning and express interest in future fertility at diagnosis [7]. The fertility challenges arising from cancer and treatments expose young women with breast cancer to multidimensional fertility concerns, including fear of infertility, anxiety about cancer recurrence associated with pregnancy, and worries about passing hereditary risk to offspring, which have been shown to significantly impact psychological well-being and quality of life [8]. Therefore, oncofertility support has emerged as an essential component of long-term breast cancer care.

Oncofertility support encompasses discussions on fertility risk, fertility preservation counseling, guidance on future fertility, information about sexual dysfunction and contraception, and fertility-related emotional and psychological support [9]. Research shows that oncofertility support empowers women to make informed decisions, provides hope, improves coping, reduces distress and decision regret, and improves quality of life [10–16]. Therefore, gaining a deeper understanding of how women with breast cancer perceive and experience oncofertility support is crucial for identifying gaps in care practices and improving oncofertility services.

However, there is no specific scale exists to measure oncofertility support. Benedict et al. have developed a five-item questionnaire to ask participants to indicate whether they have adequate information on infertility risk, early menopausal risk, fertility preservation, and family building options; however, this questionnaire lacks comprehensiveness and psychometric validation [17]. Xiao et al. later developed the Fertility Information Support Scale, which assesses fertility-related cognitive support, emotional support, and self-support related to fertility issues and demonstrates good reliability and validity [18]. Despite the scale covering various aspects of oncofertility support, such as information on fertility risk, fertility preservation, sexual health and contraception, future fertility guidance, and emotional support, it has limitations. First, the items in cognitive support dimension tend to assess participants’ knowledge (e.g., “I know there can be a family history and heredity of breast cancer”), which may not adequately capture the actual fertility information support participants received. Additionally, the scale lacks sufficient items on fertility preservation, particularly the lack of items related to fertility counseling, which has been identified as an important component in guiding reproductive decisions among women with cancer [14]. These limitations underscore the need for modifications of the scale to measure oncofertility support. This present study aimed to modify the Fertility Information Support Scale to better evaluate oncofertility support and to validate it among women with breast cancer, which is important in providing evidence to improve oncofertility care among women with breast cancer.

## Methods

### Study design

This cross-sectional study consists of two phases.

### Phase I: Development of the Oncofertility Support Scale derived from the Fertility Information Support Scale

#### Initial modification

The original Fertility Information Support Scale comprised 17 items: cognitive support (8 items); emotional support (3 items), and self-support (6 items) [18]. The research team identified a limitation in the cognitive support items, which tended to assess knowledge rather than the actual support received. Seven items were revised to more accurately reflect perceived oncofertility support. For example, the item "*I am aware of the effects of chemotherapy, endocrine therapy, and other anti-tumor treatments on fertility aspects, such as sex life and pregnancy*" was modified to "*Healthcare providers have explained the impact of anticancer treatment such as chemotherapy and endocrine therapy on fertility to me*". Furthermore, three new items on information support and one on emotional support were added, guided by international clinical guidelines [19–21], resulting in a preliminary version of the 21-item scale.

#### Further modification through expert and lay feedback

The scale was reviewed by a panel of five experts (a professor and a lecturer specializing in cancer care, a breast surgeon, and two nurses with specializations in breast cancer care) and five target participants. They evaluated the scale for necessity, relevance, and clarity, and provided suggestions for improvement. Based on feedback, two overlapping items were removed, and four new items were added, including one item addressing information support on the impact of post-cancer pregnancy on the health of women with breast cancer, one item regarding the health of their children, and two items related to fertility-related peer support. These modifications also align with the fertility information needs expressed by women with cancer [22, 23]. The revised 23-item scale was sent back to the panel for further evaluation, and no further modifications were required.

**Fig. 1:**
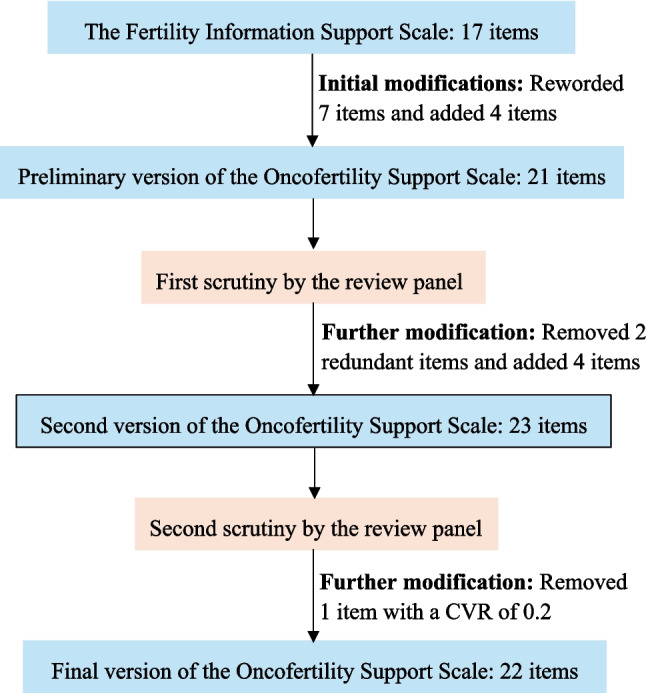
The process of the development of the Oncofertility Support Scale

The Content Validity Ratio (CVR) based on necessity and the Content Validity Index (CVI) based on relevance were calculated. Items with a CVR of ≥ 0.62 and a CVI of ≥ 0.8 were retained to ensure the scale included the most important and accurate content [24]. One item regarding peer support, with a CVR of 0.20, was removed, resulting in the final version of the scale with 22 items. The CVR for the 22-item scale ranges from 0.80 to 1.00, the item-level CVI (I-CVI) ranges from 0.90 to 1.00, and the scale-level CVI (S-CVI) is 0.95. The modification process is summarized in Fig. 1.

### Phase II: Validation of the Oncofertility Support Scale

#### Participants

The inclusion criteria were: 1) women diagnosed with breast cancer; 2) aged between 18 and 40 at diagnosis; 3) were able to understand and read Chinese; 4) no coexisting cancers.

#### Sample size calculation

Following the rule of thumb for factor analysis, the ratio of participants to variables was 5:1 for EFA and 10:1 for CFA [25]. A minimum sample of 110 for EFA and 220 for CFA was required, resulting in a required sample size of 330 participants for the study.

#### Study instruments

##### General characteristics questionnaire

A self-developed questionnaire was used to collect participants’ characteristics.

##### Oncofertility Support Scale

The Oncofertility Support Scale adapted from the Fertility Information Support Scale was used to measure oncofertility support [18]. The scale consists of 22 items, each answered using a five-point Likert scale ranging from 5 (strongly agree) to 1 (strongly disagree).

##### Medical Outcome Study (MOS) Social Support Survey

The MOS Social Support Survey is a 19-item self-report measure designed to assess four types of social support (informational/emotional, tangible, affectionate, and positive social interaction) [26]. Each item is responded with a five-point scale ranging from 1 = "None of the time" to 5 = "All of the time." Total scores range from 0 to 95, with higher scores indicating greater levels of social support. The MOS Social Support Survey has demonstrated good reliability and validity (Cronbach’s α = 0.97) [26]. The Cronbach’s α for this scale in this study is 0.97.

#### Data collection procedures

Participants were recruited via a hospital and social media (a breast cancer education and health management platform) using convenience sampling. Potential participants were initially screened to confirm eligibility. For hospital-recruited participants, diagnoses were verified through electronic medical records; for social media-recruited participants, diagnostic statuses were confirmed via the health management platform manager. Eligible participants were asked to sign an electronic informed consent form before completing a set of electronic questionnaires hosted on Wen Juan Xing.

### Ethical considerations

This study was conducted in accordance with the principles outlined in the Declaration of Helsinki and approved by the Institutional Review Boards of the University of Hong Kong/Hospital Authority Hong Kong West Cluster (UW 24–257) and the Affiliated Hospital of Guizhou Medical University (2024064K-AMD-02). All participants provided electronic consent prior to participation.

### Data analysis

The total of 343 participants was randomly split for Exploratory Factor Analysis (EFA; n1 = 123) and Confirmatory Factor Analysis (CFA; n2 = 220), in accordance with the guideline that a minimum of 220 participants is required for a reliable CFA [25]. Descriptive statistics were used to summarize participant characteristics. Chi-square (χ^2^) tests were used to examine differences in participant characteristics between the two subsamples.

EFA used principal axis factoring with Promax rotation was employed to explore the structure of the scale. The suitability of the data for the EFA was supported by the Kaiser-Meyer Olkin (KMO) test (KMO = 0.894) and Bartlett’s test of sphericity (χ^2^ = 2218, p < 0.001). Factors were retained based on eigenvalues > 1.00, and individual items with factor loadings ≥ 0.5 were retained [28].

A CFA using a Maximum Likelihood Robust (MLR) estimator was conducted with the second subsample to test the scale structure identified in the EFA, using several fit indices, including root mean square error of approximation (RMSEA ≤ 0.08), standardized root mean square residual (SRMR ≤ 0.08), comparative fit index (CFI ≥ 0.90), and Tucker-Lewis index (TLI ≥ 0.90) [29, 30].

Multigroup Confirmatory Factor Analyses were used to examine measurement invariance of the scale across groups based on age and fertility desire at survey using the full sample. First, the factor model was assessed across groups without any equality constraints. Second, metric invariance was evaluated by constraining factor loadings to be equal across groups. Third, scalar invariance was examined by additionally constraining item intercepts to be equal across groups. Fit indices, including a change in CFI (ΔCFI ≤ 0.01), RMSEA (ΔRMSEA ≤ 0.015), and SRMR (ΔSRMR ≤ 0.015 for configural and scalar invariance and ≤ 0.03 for metric invariance), were used as criteria to indicate that invariance assumptions were met [31].

Convergent validity was examined using Pearson’s correlation coefficient between the oncofertility support and medical social support [32]. Additionally, Average Variance Extraction (AVE) and Composite Reliability (CR) indicators were used to confirm the degree of convergence of the scale, with an AVE ≥ 0.50 and CR ≥ 0.70 representing acceptable convergent validity [33].

Known-group validity was assessed by comparing participants based on age at survey (≤ 35 vs. > 35) and fertility desire at survey (certain/uncertain vs. no fertility desire). It was hypothesized that younger participants and those expressing a desire for fertility would receive more oncofertility support [34, 35, 37]. Cohen’s d effect sizes were calculated [32].

Reliability was assessed using internal consistency via both Cronbach’s α and McDonald’s *ω* coefficient. A reliability coefficient > 0.80 is considered adequate [38]. Internal construct validity was estimated using the corrected item-total correlation, with a threshold of 0.4 set for adequate correlations [39].

Data was analyzed using IBM SPSS (version 29.0) and Mplus (version 8.3), and a p-value of < 0.05 was considered statistically significant.

## Results

### Demographic characteristics of participants

From August to September 2024, 406 women with breast cancer accessed the online survey, with 343 completed the survey. Among 63 who did not complete the survey, five did not click the consent button to continue the survey, 34 did not pass the eligibility screening, and 24 passed the eligibility screening but did not complete the survey. The mean current age of participants was 34.32 (SD = 4.48), ranging from 20 to 45. Other characteristics of the participants are presented in Table 1. No significant differences were found between subsamples.

**Table 1:** Demographic Characteristics of Participants

Characteristics	Total sample (*N* = 343)	Subsample one (n1 = 123)	Subsample two (n2 = 220)
	*n* (%)		
Age at diagnosis 32.54 (SD = 4.33) ≤ 35 > 35	255 (74.34) 88 (25.66)	89 (72.36) 34 (27.64)	166 (75.45) 54 (24.55)
Age at survey 34.32 (SD = 4.48) ≤ 35 > 35	196 (57.14) 147 (42.86)	72 (58.54) 51 (41.46)	124 (56.36) 96 (43.64)
Marital status Married Unmarried/Divorced/Widowed	245 (71.43) 98 (28.57)	84 (68.29) 39 (31.71)	161 (73.18) 59 (26.82)
Chinese ethnics Han Minority	329 (95.92) 14 (4.08)	115 (93.50) 8 (6.50)	214 (97.27) 6 (2.73)
Educational level Under bachelor Bachelor and above	133 (38.78) 210 (61.22)	42 (34.15) 81 (65.86)	91 (41.36) 129 (58.64)
Employment status Yes No	236 (68.80) 107 (31.20)	90 (73.17) 33 (26.83)	146 (66.36) 74 (33.64)
Family monthly income per person (RMB) < 5000 5000—10,000 > 10,000	132 (38.78) 159 (46.06) 52 (15.16)	45 (36.59) 61 (49.59) 17 (13.82)	87 (39.55) 98 (44.55) 35 (15.90)
Parenthood status at diagnosis No children ≥ 1 child	143 (41.69) 200 (58.31)	56 (45.53) 67 (54.47)	87 (39.55) 133 (60.45)
Fertility desire at diagnosis Yes/Uncertain No	169 (49.27) 174 (50.73)	58 (47.15) 65 (52.85)	111 (50.45) 109 (49.55)
Fertility desire at survey Yes/Uncertain No	141 (41.11) 202 (58.89)	49 (39.84) 74 (60.16)	92 (41.82) 128 (58.18)
Cancer stage Stage 0-II Stage III-IV	262 (76.38) 81 (23.62)	93 (75.61) 30 (24.39)	169 (76.82) 51 (23.18)
Time since diagnosis ≤ 2 years > 2 years	237 (69.10) 106 (30.90)	92 (74.80) 31 (25.20)	145 (65.91) 75 (34.09)
Surgery Yes No	337 (98.25) 6 (1.75)	120 (97.56) 3 (2.44)	217 (98.64) 3 (1.36)
Chemotherapy Yes No	312 (90.96) 31 (9.04)	113 (91.87) 10 (8.13)	199 (90.45) 21 (9.55)
Radiotherapy Yes No	261 (76.09) 82 (23.91)	94 (76.42) 29 (23.58)	167 (75.91) 53 (24.09)
Endocrine therapy Yes No	267 (77.84) 76 (22.16)	91 (73.98) 32 (26.02)	176 (80.00) 44 (20.00)
Target therapy Yes No	116 (33.82) 227 (66.18)	40 (32.52) 83 (67.48)	76 (34.55) 144 (65.45)
Whether have completed all treatment Yes No	154 (44.90) 189 (55.10)	59 (47.97) 64 (51.03)	95 (43.18) 125 (56.82)

*N* the overall sample size, *n1* subsample one, *n2* subsample two

### Exploratory factor analysis with subsample one

The initial EFA revealed a four-factor structure, with eigenvalues > 1.0. The initial item distribution was as follows: Factor 1 comprised four items, Factors 2 and 3 included five items each, and Factor 4 consisted of eight items. Two items (Item 8 on Factor 3 and Item 13 on Factor 2) with factor loadings below 0.5 were removed, leading to a second EFA. In the second EFA, Factors 2 and 3 each retained four items. Item 14 on Factor 2, with a factor loading 0.485, was also removed before conducting the final EFA. The final EFA resulted in a 19-item scale with four factors: Factor 1 ("Information Support on Fertility Impact") with four items, Factor 2 ("Information Support on Fertility Preservation") with three items, Factor 3 ("Fertility and Sexual Guidance") with four items, and Factor 4 ("Fertility Communication and Supportive Networks") with eight items. This structure accounted for 75.77% of the total variance (Table 2).

**Table 2:** Item factor loadings from the exploratory factor analysis with subsample one (*n* = 123)

Subscale/Items	Factor			
	Factor 1	Factor 2	Factor 3	Factor 4
Informational support on fertility impact				
1. Healthcare providers have explained the impact of chemotherapy and endocrine therapy on fertility to me	**0.703**			
2. Healthcare providers have provided information about the family history and heredity of breast cancer to me	**0.865**			
3. Healthcare providers have provided me with information on the impact of pregnancy after breast cancer on my health	**0.868**			
4. Healthcare providers have given me information about the impact of breast cancer treatment on the health of children	**0.940**			
Informational support on fertility preservation				
5. Healthcare providers have introduced fertility preservation methods to me, such as medication, embryo cryopreservation, egg cryopreservation, or ovarian tissue cryopreservation		**0.998**		
6. Healthcare providers have explained the safety, benefits, and risks of fertility preservation strategies to me		**0.792**		
7. Healthcare providers have informed me that I can have a fertility consultation with fertility specialists if needed		**0.812**		
Fertility and sexual guidance				
9. Healthcare providers have offered me information on breastfeeding after breast cancer treatment			**0.563**	
10.The healthcare providers have informed me about ways to improve my sexual life, such as using lubricants and strengthening pelvic floor exercises			**0.888**	
11. Healthcare providers have provided me with information about suitable contraception methods, such as condoms and vaginal diaphragms			**1.021**	
12. I have received a professional fertility information handbook (either in paper or electronic) from healthcare providers			**0.817**	
Fertility communication and support network				
15. My family (especially my spouse) is able to work with and support me in dealing with my fertility issues				**0.563**
16. I have access to communicate with peers and share psychological distress related to fertility				**0.566**
17. I can actively talk to my family (spouse) about my childbearing intentions				**0.762**
18. I am able to talk to my healthcare provider about my childbearing needs				**0.701**
19. I am able to proactively talk with my healthcare provider about my fertility confusion				**0.778**
20. I am very aware of my fertility needs and the possible risks of childbirth				**1.007**
21. I am able to take the initiative to obtain fertility information in other ways, such as books and the internet				**0.977**
22. When I am worried about my fertility, I am able to adapt myself psychologically or seek outside help				**0.964**

no items cross-load onto another factor with factor loading ≥ 0.3

### Confirmatory factor analysis with subsample two

**Fig. 2:**
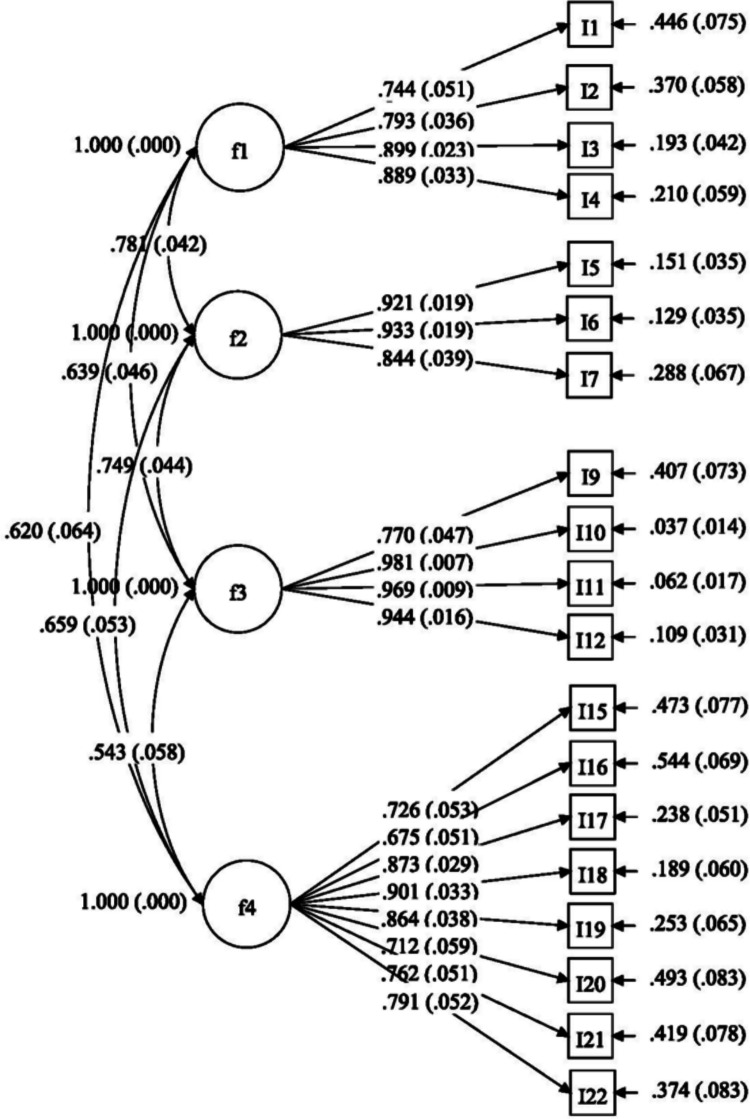
Final model of oncofertility support (*n* = 220)

The CFA results confirmed the four-factor structure as acceptable (χ^2^/df = 2.37; RMSEA = 0.079; SRMR = 0.060; CFI = 0.914; TLI = 0.900). All 19 items loaded significantly onto their respective factors, with standardized loadings ranging from 0.68 to 0.98 (Fig. 2).

#### Measurement invariance

##### Measurement invariance across age groups

First, the four-factor model was tested across two age groups (age at survey: ≤ 35 and > 35) without any equality constraints. The model fit indices were: χ^2^ = 609.478, df = 292, CFI = 0.913, TLI = 0.898, SRMR = 0.066, RMSEA = 0.080 (90% CI: 0.071–0.088).

To test metric invariance, equal factor loadings were introduced. This resulted in a slight decrease in model fit: χ^2^ = 639.001, df = 307, CFI = 0.909, TLI = 0.899, SRMR = 0.073, RMSEA = 0.079 (90% CI: 0.071–0.088), ΔCFI = 0.004, ΔSRMR = 0.007, ΔRMSEA = 0.001.

For scalar invariance, equal item intercepts were added. This model showed a further decrease in fit: χ^2^ = 662.727, df = 322, CFI = 0.907, TLI = 0.901, SRMR = 0.075, RMSEA = 0.079 (90% CI: 0.070–0.087), ΔCFI = 0.002, ΔSRMR = 0.002, ΔRMSEA = 0.000.

##### Measurement invariance across fertility desire groups

Configural invariance was also tested across fertility desire at survey groups (certain/uncertain fertility desire vs no fertility desire at survey). The model fit indices were: χ^2^ = 632.384, df = 292, CFI = 0.905, TLI = 0.889, SRMR = 0.072, RMSEA = 0.082 (90% CI: 0.074–0.091).

When equal factor loadings were imposed to test for metric invariance, the model fit indices showed a decrease: χ^2^ = 646.737, df = 307, CFI = 0.905, TLI = 0.894, SRMR = 0.073, RMSEA = 0.080 (90% CI: 0.072–0.089), ΔCFI = 0.000, ΔSRMR = 0.001, ΔRMSEA = 0.002.

The model for scalar invariance also showed a reduction in model fit compared to the metric model, with χ^2^ = 686.793, df = 322, CFI = 0.898, TLI = 0.892, SRMR = 0.074, RMSEA = 0.081 (90% CI: 0.073–0.090), ΔCFI = 0.007, ΔSRMR = 0.001, ΔRMSEA = 0.001 (Table 3).

**Table 3:** Model fit indices for testing the measurement invariance across groups based on age and fertility desire at survey (*N* = 343)

	χ^2^	df	CFI	TLI	SRMR	RMSEA (90% CI)	ΔCFI	ΔRMSEA	ΔSRMR
Age at currently: Group 1 = ≤ 35 (*n* = 196); Group 2 = > 35 (*n *= 147)									
Configural invariance	609.478	292	0.913	0.898	0.066	0.080 (0.071–0.088)	-	-	-
Metric invariance	639.001	307	0.909	0.899	0.073	0.079 (0.071–0.088)	0.004	0.001	0.007
Scalar invariance	662.727	322	0.907	0.901	0.075	0.079 (0.070–0.087)	0.002	0.000	0.002
Fertility desire at currently: Group 1 = Yes/Uncertain (*n* = 141); Group 2 = No (*n* = 202)									
Configural invariance	632.384	292	0.905	0.889	0.072	0.082 (0.074–0.091)	-	-	-
Metric invariance	646.737	307	0.905	0.894	0.073	0.080 (0.072–0.089)	0.000	0.002	0.001
Scalar invariance	686.793	322	0.898	0.892	0.074	0.081 (0.073–0.090)	0.007	0.001	0.001

### Convergent validity

The information support on fertility impact (r = 0.17, p = 0.001), fertility communication and supportive network (r = 0.25, p < 0.001), and the total oncofertility support (r = 0.18, p < 0.001) show significant correlations with the total medical social support (Table 4).

**Table 4:** Correlations between oncofertility support and medical social support (*N* = 343)

The Oncofertility Support Scale	r	p
Information support on fertility impact	0.17	0.001
Information support on fertility preservation	0.09	0.107
Fertility and sexual guidance	0.04	0.477
Fertility communication and supportive network	0.25	< 0.001
Total score	0.18	< 0.001

The AVE for the four dimensions ranged from 0.63 to 0.85, and the CR ranged from 0.90 to 0.96, supporting the scale’s convergent validity (Table 5).

**Table 5:** AVE and CR values for each dimension

Dimension	Number of items	AVE	CR
Information support on fertility impact	4	0.70	0.90
Information support on fertility preservation	3	0.81	0.93
Fertility and sexual guidance	4	0.85	0.96
Fertility communication and supportive network	8	0.63	0.93

### Known-group validity

Known-group validity analysis using the full sample revealed that younger women (≤ 35 years) reported higher scores on information support on fertility preservation (Cohen’s d = 0.28, p = 0.010) and fertility communication and supportive networks (Cohen’s d = 0.27, p = 0.014). No significant differences were found in overall oncofertility support or other dimensions between age groups. Additionally, women without fertility desire scored higher on fertility and sexual guidance compared to those with fertility desire or uncertainty (Cohen’s d = 0.26, p = 0.019). No significant differences were observed in overall oncofertility support or other dimensions between fertility desire groups (Table 6).

**Table 6:** Known-group validity of the scale (*N* = 343)

	Mean (SD)		t	p	Absolute Cohen’s d
	^*^ ≤ 35 (*n* = 196)	^*^ > 35 (*n* = 147)			
Information support on fertility impact	3.73 (1.01)	3.58 (1.10)	1.34	0.181	0.15
Information support on fertility preservation	3.29 (1.26)	2.94 (1.22)	2.59	0.010	0.28
Fertility and sexual guidance	2.58 (1.25)	2.65 (1.24)	-0.49	0.625	0.05
Fertility communication and supportive networks	3.75 (0.89)	3.50 (0.95)	2.47	0.014	0.27
Total score	3.42 (0.87)	3.25 (0.91)	1.83	0.068	0.20
	^#^Yes/Uncertain (*n* = 169)	^#^Without (*n* = 174)			
Information support on fertility impact	3.59 (1.01)	3.72 (1.07)	-1.09	0.278	0.12
Information support on fertility preservation	3.19 (1.20)	3.11 (1.29)	0.61	0.541	0.07
Fertility and sexual guidance	2.42 (1.18)	2.74 (1.27)	-2.36	0.019	0.26
Fertility communication and supportive networks	3.62 (0.87)	3.66 (0.96)	-0.35	0.728	0.04
Total score	3.29 (0.82)	3.39 (0.94)	-0.97	0.332	0.11

^*^Age at survey; ^#^Fertility desire at survey

### Reliability

Cronbach’s α and McDonald’s ω coefficients were calculated using the full sample. The Cronbach’s α for the total scale was 0.95, with subscale values ranging from 0.90 to 0.95. Corrected item-total correlations ranged from 0.62 to 0.78. McDonald’s ω for the total scale was 0.95, with subscale values ranging from 0.91 to 0.95 (Supplementary table S1).

## Discussion

This study validated the Oncofertility Support Scale as a reliable and valid tool for evaluating oncofertility support.

The EFA identified a four-factor structure with 19 items: information support on fertility impact, information support on fertility preservation, fertility and sexual guidance, and fertility communication and supportive networks. The informational support on fertility impact focuses on information about the effects of cancer treatment on fertility and the health implications of post-diagnosis fertility for women and their children. This aligns with findings from Goossens et al. (2014), which highlighted that 66% to 100% of patients with cancer need information on treatment-related fertility impacts, as well as potential risks to offspring[22]. The information support on fertility preservation dimension emphasizes the importance of providing information on fertility preservation options. Previous research indicates that awareness of these options helps reduce emotional distress and fosters optimism about life after cancer [12, 40]. Both dimensions align with the American Society of Clinical Oncology’s recommendations, which advocate for integrating fertility risk assessments and preservation discussions into cancer care for women with breast cancer [21].

In contrast to the information on fertility impact and preservation, which are crucial before treatment for informed decision-making. The fertility and sexual guidance dimension focuses on information relevant during follow-up, addressing ongoing concerns such as breastfeeding, sexual dysfunction, and contraception [16, 23, 41]. Mesters et al. noted that patients’ information needs shift from information about disease and treatment to practical guidance during follow-up [42]. The final dimension focuses on fertility communication and supportive networks, emphasizing the importance of discussing fertility issues with family, spouses, and peers for emotional support and decision-making [43]. This dimension reflects how women with breast cancer perceive and experience support in navigating fertility-related challenges.

Based on the EFA results, three items (8, 13, 14) with factor loadings ≤ 0.50 were excluded. Item 8, which addressed the timing of pregnancy, was removed. This finding contrasts with previous research showing that women with breast cancer often want information on pregnancy timing. The discrepancy may be due to differences in participant groups. The previous study focused on women who had given birth after treatment [23], while most participants in this study were still undergoing treatment, making pregnancy timing a less immediate concern. Furthermore, decisions about pregnancy timing are highly individualized, influenced by treatment type, recovery, and health monitoring [42]. Therefore, pregnancy timing may not have been perceived as urgent or relevant support needs by participants. Items 13 and 14, related to emotional support from healthcare professionals, were also excluded. This aligns with a qualitative study showing that Chinese women with breast cancer seek fertility information from healthcare professionals but rely more on family, especially partners, for emotional support [23]. The absence of roles like social workers or oncofertility navigators in the Chinese healthcare system, which are common in Western countries [16, 43], may explain the lower expectations for emotional support from healthcare professionals.

The acceptable fit indexes (RMSEA = 0.079; SMRM = 0.060; CFI = 0.914; TLI = 0.900) support the four-factor model. Furthermore, measurement invariance across age and fertility desire groups was confirmed, indicating that the factor structure, the relationships between items and their corresponding factors, and the interpretation and use of the response scale were consistent across these groups. However, the marginal fit indices suggest that future studies with larger samples are needed to further validate these findings.

The observed correlations between medical social support and oncofertility support (e.g., total oncofertility support, information about fertility impact, and communication/supportive networks) were significant but weak. In contrast, information support on fertility preservation and fertility and sexual guidance showed no significant associations with medical social support. This finding may be attributed to the broader nature of medical social support [26], which does not capture fertility-related support in patients with cancer. Future research should consider selecting more specific measures that better align with the unique aspects of oncofertility support to further establish the convergent validity of the Oncofertility Support Scale.

The analysis of known-group validity revealed significant differences in information support on fertility preservation and fertility communication and supportive network across age groups. This finding aligns with previous research indicating that healthcare providers are more likely to offer oncofertility care to younger patients [34]. However, contrary to prior studies indicating that healthcare providers tend to offer more support to women who express a desire for children [35], no significant differences of oncofertility support were observed across fertility desire groups. This discrepancy may stem from heightened expectations among women who desire children, leading them to perceive the support they receive as inadequate.

Finally, the Oncofertility Support Scale shows strong reliability, with Cronbach’s alpha and McDonald’s ω values for the total scale and all four dimensions exceeding 0.90. Item-total correlations above 0.60 further confirm that individual items are strongly related to the overall scale score.

## Limitations

Several limitations should be noted. First, the study did not include structured involvement of patient advocacy organizations (PAOs) during the development phase due to resource constraints. The absence of stakeholder participation may have restricted the breadth of perspectives incorporated into the scale and thus may have affected content validity. Future research should integrate PAOs more systematically to ensure that the scale reflects the full range of oncofertility support needs among young women with breast cancer. Second, the sample comprised Chinese-speaking participants recruited from a single hospital and one social media platform, which limits the external validity of the findings. In the Chinese cultural context, women with breast cancer often prioritize emotional support from family members while relying on healthcare providers primarily for informational and decision-making support [23]. Such preferences may differ in other sociocultural settings. To enhance cross-cultural applicability, future studies should include non-Chinese participants and employ forward–backward translation and differential item functioning analyses to evaluate potential cultural differences in perceptions of oncofertility support. Third, the study did not assess test–retest reliability, which is essential for evaluating the scale’s stability over time. Future assessments of the scale should incorporate this to establish its reliability. Finally, BRCA1/2 mutation status was not collected, preventing identification of participants with hereditary breast cancer. As BRCA carriers typically require more intensive and personalized fertility counseling, the absence of genetic information limits the ability to determine whether the scale adequately captures the unique needs of this subgroup. Future research should validate the scale among women with hereditary breast cancer and examine whether oncofertility support varies by BRCA mutation status.

## Conclusion

This study establishes the Oncofertility Support Scale as a valid and reliable measure for evaluating oncofertility support among young women with breast cancer. The scale can guide the development of targeted interventions to improve oncofertility services among women with breast cancer.

## Supplementary Information

Below is the link to the electronic supplementary material.

ESM 1 (DOC 58.0 KB)

## Data Availability

The data supporting the findings of this study are available from the corresponding author upon reasonable request.
