# Supplementary material for: Validation of the Oncofertility Support Scale derived from the Fertility Information Support scale among young adult women with breast cancer
Source: Support Care Cancer. 2025 Dec 19;34(1):48. doi: 10.1007/s00520-025-10250-0 (PMC12717119; doi:10.1007/s00520-025-10250-0)
Supplement: Supplementary file 1 — (DOC 58.0 KB) [file 520_2025_10250_MOESM1_ESM.doc]

**Supplementary Table S1 Reliability of the scale (N= 343)**

|  | Score = 5 | Score = 4 | Score = 3 | Score = 2 | Score = 1 | Cronbach’s α | McDonald’s ω | Corrected item-total correlation |
| --- | --- | --- | --- | --- | --- | --- | --- | --- |
|  | n (%) | n (%) | n (%) | n (%) | n (%) |  |  |  |
| **Dimension 1: Information Support on Fertility Impact** | | | | | | 0.90 | 0.91 |  |
| Item 1 | 128 (37.32) | 52 (15.16) | 132 (38.48) | 22 (6.41) | 9 (2.62) |  |  | 0.67 |
| Item 2 | 145 (42.27) | 52 (15.16) | 108 (31.49) | 21 (7.87) | 11 (3.21) |  |  | 0.62 |
| Item 3 | 117 (34.11) | 44 (12.83) | 115 (33.53) | 51 (14.87) | 16 (4.66) |  |  | 0.72 |
| Item 4 | 108 (31.49) | 41 (11.95) | 116 (33.82) | 54 (15.74) | 24 (7.00) |  |  | 0.76 |
| **Dimension 2: Information Support on Fertility Preservation** | | | | | | 0.92 | 0.92 |  |
| Item 5 | 81 (23.62) | 37 (10.79) | 88 (25.66) | 88 (25.66) | 49 (14.29) |  |  | 0.73 |
| Item 6 | 83 (24.20) | 37 (10.79) | 104 (30.32) | 77 (22.45) | 83 (24.20) |  |  | 0.78 |
| Item 7 | 98 (28.57) | 34 (9.91) | 108 (31.49) | 67 (19.53) | 36 (10.50) |  |  | 0.75 |
| **Dimension 3: Fertility and Sexual Guidance** | | | | | | 0.95 | 0.95 |  |
| Item 9 | 62 (18.08) | 21 (6.12) | 81 (23.62) | 116 (33.82) | 63 (18.37) |  |  | 0.74 |
| Item 10 | 55 (15.03) | 20 (5.83) | 75 (21.87) | 114 (33.24) | 79 (23.03) |  |  | 0.71 |
| Item 11 | 54 (15.74) | 20 (5.83) | 84 (24.49) | 108 (31.49) | 77 (22.45) |  |  | 0.70 |
| Item 12 | 55 (16.03) | 18 (5.25) | 63 (18.37) | 124 (36.15) | 83 (24.20) |  |  | 0.70 |
| **Dimension 4: Fertility Communication and Supportive Network** | | | | | | 0.94 | 0.94 |  |
| Item 15 | 120 (34.99) | 43 (12.54) | 135 (39.36) | 30 (8.75) | 15 (4.37) |  |  | 0.65 |
| Item 16 | 104 (30.32) | 47 (13.70) | 134 (39.07) | 43 (12.54) | 15 (4.37) |  |  | 0.65 |
| Item 17 | 106 (30.90) | 49 (14.29) | 136 (39.65) | 42 (12.24) | 10 (2.92) |  |  | 0.71 |
| Item 18 | 98 (28.57) | 50 (14.58) | 134 (39.07) | 47 (13.70) | 14 (4.08) |  |  | 0.75 |
| Item 19 | 108 (31.49) | 57 (16.62) | 136 (39.65) | 32 (9.33) | 10 (2.92) |  |  | 0.71 |
| Item 20 | 122 (35.57) | 53 (15.45) | 134 (39.07) | 28 (8.16) | 6 (1.75) |  |  | 0.62 |
| Item 21 | 123 (35.86) | 54 (15.74) | 146 (42.57) | 15 (4.37) | 5 (1.46) |  |  | 0.67 |
| Item 22 | 112 (32.65) | 45 (13.12) | 157 (45.77) | 21 (6.12) | 8 (2.33) |  |  | 0.62 |
| **Overall scale** | | | | | | 0.95 | 0.95 |  |
